# SncRNA715 Inhibits Schwann Cell Myelin Basic Protein Synthesis

**DOI:** 10.1371/journal.pone.0136900

**Published:** 2015-08-28

**Authors:** Christina Müller, Nina M. Hochhaus, Xavier Fontana, Heiko J. Luhmann, Robin White

**Affiliations:** 1 Institute of Physiology, University Medical Center of the Johannes Gutenberg University, Mainz, Germany; 2 MRC Laboratory for Molecular Cell Biology and the UCL Cancer Institute, University College London, London, United Kingdom; University of Muenster, GERMANY

## Abstract

Myelin basic proteins (MBP) are major constituents of the myelin sheath in the central nervous system (CNS) and the peripheral nervous system (PNS). In the CNS *Mbp* translation occurs locally at the axon-glial contact site in a neuronal activity-dependent manner. Recently we identified the small non-coding RNA 715 (sncRNA715) as a key inhibitor of *Mbp* translation during transport in oligodendrocytes. *Mbp* mRNA localization in Schwann cells has been observed, but has not been investigated in much detail. Here we could confirm translational repression of *Mbp* mRNA in Schwann cells. We show that sncRNA715 is expressed and its levels correlate inversely with MBP in cultured Schwann cells and in the sciatic nerve *in vivo*. Furthermore we could reduce MBP protein levels in cultured Schwann cells by increasing the levels of the inhibitory sncRNA715. Our findings suggest similarities in sncRNA715-mediated translational repression of *Mbp* mRNA in oligodendrocytes and Schwann cells.

## Introduction

In the peripheral nervous system myelinating Schwann cells form a lipid-rich myelin membrane around axonal segments allowing saltatory conduction of action potentials. Proliferation, migration and myelination of Schwann cells is controlled by the neuronal EGF-receptor family protein Neuregulin 1 (NRG1) which binds to Schwann cell ErbB2/3 receptors and activates second messenger cascades [1–5]. Upon this interaction myelination takes place very locally suggesting spatial and temporal regulatory mechanisms [6,7].

One of the major myelin proteins in the CNS as well as in the PNS is Myelin Basic Protein (MBP) [7]. Its absence results in severe hypomyelination in the CNS while no defects in myelin thickness and compaction are observable in the PNS [8,9] where the P0 protein seems to compensate major dense line deficits [10]. However, the numbers of Schmidt-Lantermann incisures (SLI) are increased in the sciatic nerve of *shiverer* mice lacking functional MBP [11]. Apparently, Schwann cell MBP controls these numbers by affecting the stability and turnover rate of SLI proteins such as Connexin-32 and Myelin Associated Glycoprotein (MAG). The expression of both proteins is inversely proportional to MBP in the sciatic nerve of *shiverer* mice [12].

During the myelination process in the PNS *Mbp* mRNA can be found diffusely distributed throughout the cytoplasm of the myelinating Schwann cell and localized transport and translational inhibition is suggested [13]. It was shown by *in situ* hybridization in fixed teased fibers of the sciatic nerve that *Mbp* mRNA is focally concentrated at paranodal areas in addition to having a more diffuse pattern along the internode [14]. Oligodendroglial *Mbp* mRNA is transported in a translationally silenced state to the axon-glial contact site in RNA granules. This transport depends on binding of the trans-acting factor heterogeneous nuclear ribonucleoprotein (hnRNP) A2 to the A2 response element (A2RE) in the 3’UTR of *Mbp* mRNA [15]. One major regulator of oligodendroglial *Mbp* translation is the 21nt long small non-coding RNA 715 (sncRNA715) which acts directly on a specific region of *Mbp* mRNAs 3’UTR and inhibits its translation [16]. It is not known if sncRNA715 is expressed by Schwann cells and if *Mbp* translation is regulated by this small regulatory RNA.

Recent studies have emphasized the roles of small non-coding RNAs (sncRNAs) in the regulation of myelination in the PNS. For instance miRNA-29a regulates the expression of PMP22, a major component of compact myelin, and miRNA-138 controls the transcription factor Sox2 which is expressed by immature Schwann cells and repressed during differentiation [17,18]. Schwann cells lacking the sncRNA-processing enzyme Dicer lose their ability to produce myelin [17,19,20].

Here we analyzed if sncRNA715 regulates MBP synthesis in Schwann cells. We show the expression of sncRNA715 in Schwann cells and demonstrate the inverse correlation of *Mbp* mRNA and sncRNA715 in cultured cells and the sciatic nerve. Furthermore we confirm the inhibitory effect of sncRNA715 on MBP in differentiating primary Schwann cells suggesting a role of sncRNA715 as a key regulator of MBP synthesis in the PNS similar to its role in the CNS.

## Results

### MBP is translationally repressed in IMS32 cells

**Fig 1:**
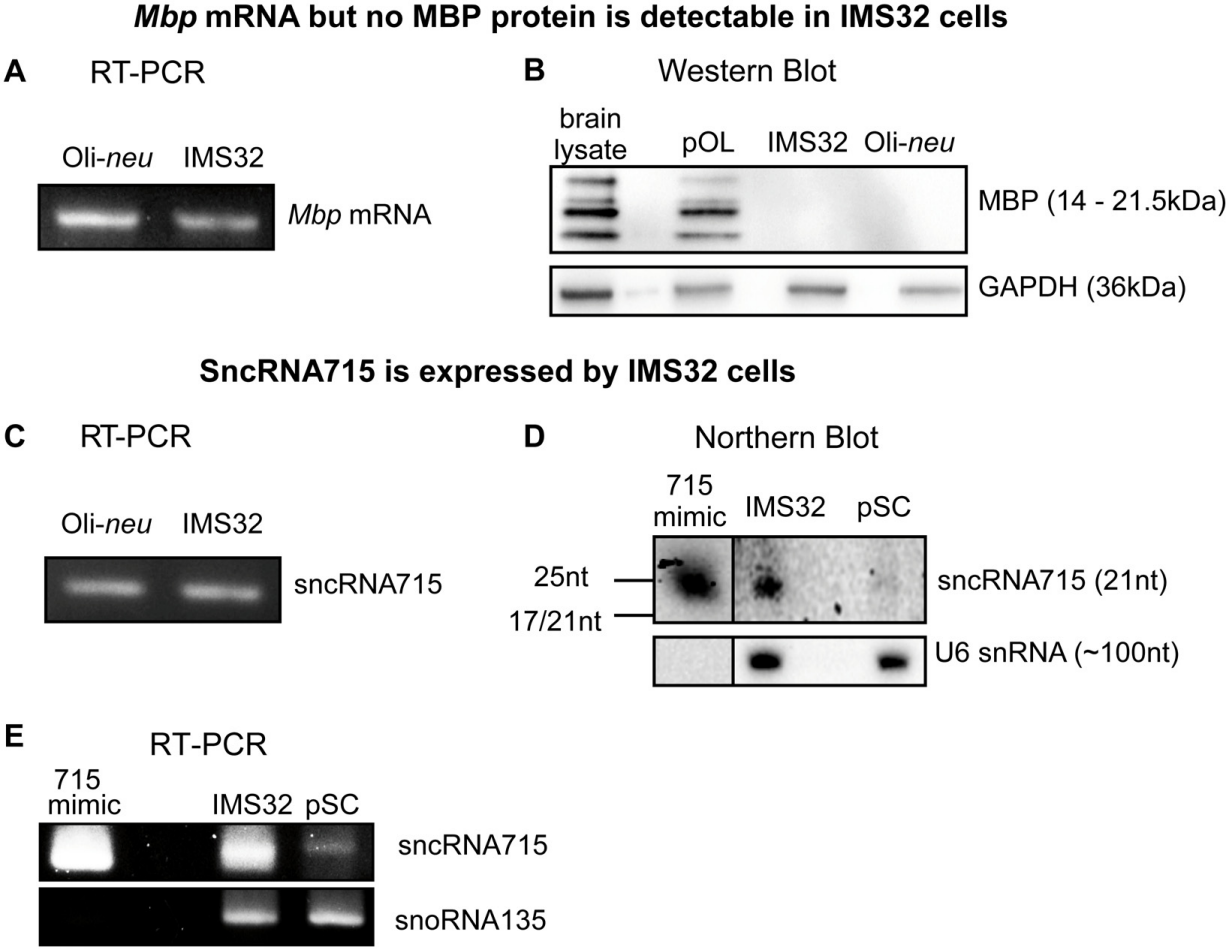
MBP and sncRNA715 Expression in Schwann cells. **A**, Reverse transcription PCR (RT-PCR) on RNA extracted from Oli-*neu* or IMS32 cells using *Mbp*-specific primers. The 88nt long amplicon for *Mbp* was visualized in an ethidium bromide-stained 4% agarose gel. **B**, Western Blots of lysates from P18 mouse brain (brain lysate), primary oligodendrocytes (pOL, 7DIV), IMS32 and Oli-*neu* cells using MBP and GAPDH (loading control) specific antibodies. **C**, Reverse transcription PCR (RT-PCR) on RNA extracted from Oli-*neu* or IMS32 cells using a sncRNA715-specific primer assays. PCR products (~60-nt long due to the use of hairpin primers in the RT reaction) were visualized in an ethidium bromide stained 4% agarose gel. **D**, Northern Blots with RNA from IMS32 and undifferentiated primary Schwann cells (pSC) shows expression of sncRNA715 in IMS32 and a lower expression in pSC. Synthetic sncRNA715 (715-mimic) and U6 snRNA were used as positive control and loading control, respectively. **E**, RT-PCR on RNA from IMS32 and undifferentiated pSC confirms lower expression of sncRNA715 in pSC compared to IMS32 cells shown in D. 715-mimic was used as positive control and snoRNA135 as loading control.

Oligodendrocyte progenitor cells (OPCs) as well as the OPC line Oli-*neu* contain *Mbp* mRNA, high levels of the inhibitory sncRNA715 and lack MBP protein [16]. We initially addressed the questions if undifferentiated Schwann cells contain *Mbp* mRNA while also lacking MBP protein, to assess if *Mbp* mRNA is translationally repressed in these cells as well. We extracted total RNA and proteins from the spontaneously immortalized murine Schwann cell line IMS32 [21]. Reverse transcription and subsequent PCR (RT-PCR) with MBP-specific primers revealed the presence of *Mbp* mRNA in these cells similar to Oli-*neu* cells which we used as a positive control (Fig 1A) whereas a water control did not show any signal (data not shown). Western Blot analysis with MBP-directed antibodies showed that both Oli-*neu* cells as well as IMS32 cells do not contain detectable MBP protein in contrast to differentiated cultured primary oligodendrocytes (7 days *in vitro*, DIV) and 18 day old mouse brains (Fig 1B). Glyceraldehyde 3-phosphate dehydrogenase (GAPDH) served as a loading control in these experiments. The presence of *Mbp* mRNA and absence of MBP proteins suggests that *Mbp* translation is also inhibited in the IMS32 cell line.

We then checked for sncRNA715 expression in these cells and found by RT-PCR that IMS32 contain this small regulatory RNA like Oli-*neu* cells which served as a positive control (Fig 1C) and RT-PCR on water as negative control did not result in any signal (data not shown). We also performed northern blots and RT-PCR with RNA isolated from IMS32 and primary cultured Schwann cells and detected sncRNA715 and the control spliceosomal U6 RNA (U6 snRNA) or snoRNA135, respectively. In these experiments we used the synthetic 715-mimic RNA as a positive control which has the same sequence as endogenous sncRNA715. The Northern Blot for sncRNA715 proves expression of sncRNA715 in IMS32 cells and a lower expression also in primary Schwann cells (pSC) revealed by a faint band in the blot (Fig 1D) which could be confirmed by RT-PCR (Fig 1E).

Given the known function of sncRNA715 in the translational inhibition of *Mbp* in the CNS, these findings together suggest the same function of sncRNA715 in Schwann cells.

### *Mbp* protein and sncRNA715 inversely correlate in differentiating Schwann cells

The expression of sncRNA715 should be downregulated to allow MBP protein synthesis if this regulatory RNA inhibits Schwann cell *Mbp* translation. We therefore analyzed if sncRNA715 levels decrease in cultured differentiated Schwann cells which contain MBP protein.

**Fig 2:**
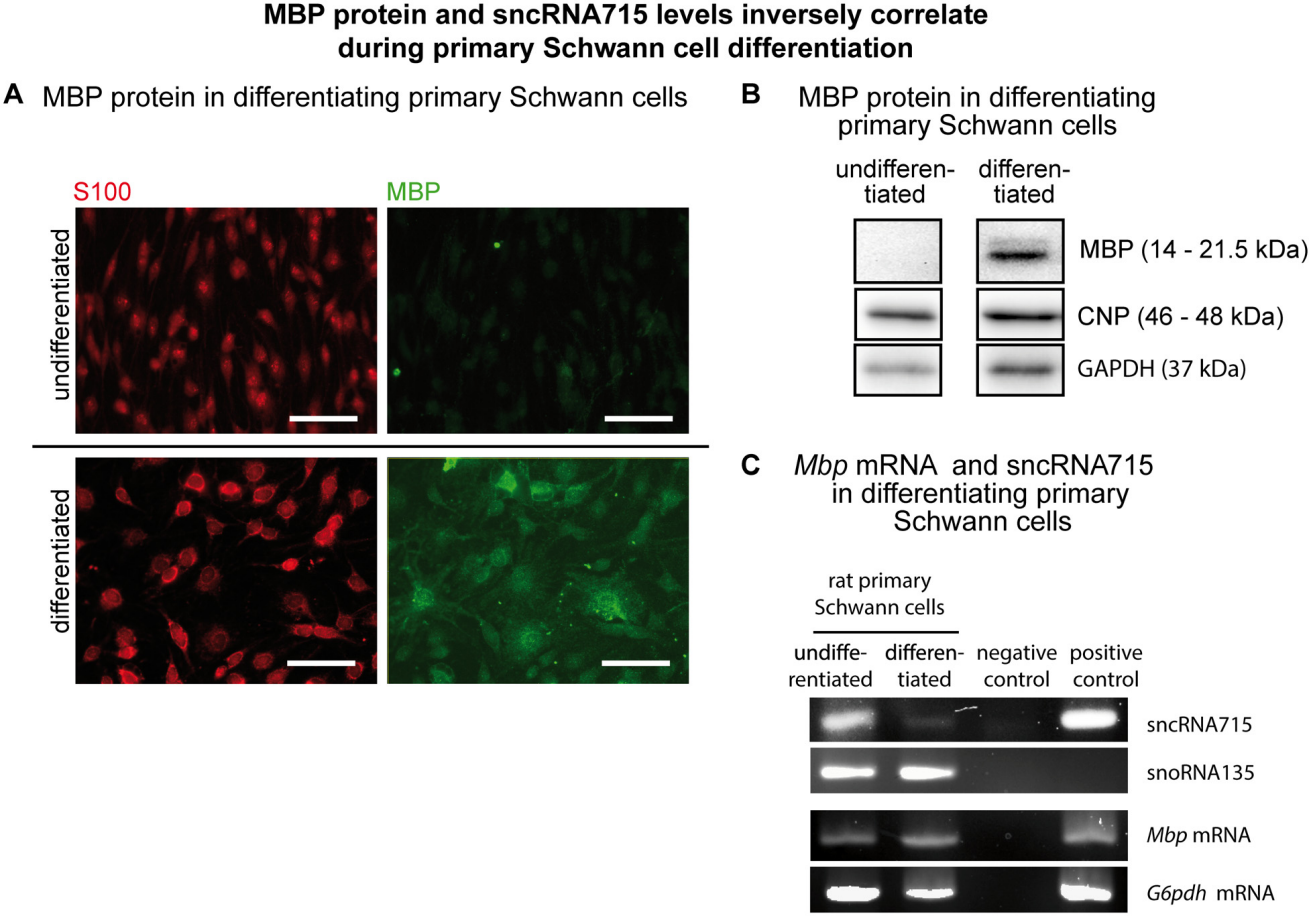
Inverse correlation of MBP and sncRNA715 in primary Schwann cells. **A**, Primary Schwann cells derived from sciatic nerves of P3 Wistar rats were cultured in non-differentiating (untreated) or differentiating (+NRG1 +dbcAMP) conditions. MBP protein can only be detected by immunocytochemistry in differentiated Schwann cells. Scale bar represents 50μm. **B**, Western Blots of undifferentiated and differentiated primary Schwann cells show MBP protein only present in differentiated Schwann cells. CNP is expressed in both maturation stages of primary Schwann cells. GAPDH serves as loading control. **C**, MBP and sncRNA715-specific RT-PCR on RNA extracted from undifferentiated or differentiated primary Schwann cells. *Mbp* mRNA is present at both differentiation states while sncRNA715 is detectable in undifferentiated and hardly in differentiated Schwann cells. SnoRNA135 and *G6pdh* mRNA were used as loading controls.

We differentiated cultured primary Schwann cells by the addition of NRG1 and dibutyryl-cAMP (dbcAMP) [5,22] and confirmed MBP protein synthesis in those cells by immunocytochemistry and Western blotting. In undifferentiated S100-positive Schwann cells MBP protein is not detectable whereas differentiated cells show immunoreactivity for MBP (Fig 2A). Similarly, Western Blot analysis reveals no MBP protein in undifferentiated Schwann cells in contrast to differentiated cells in which MBP protein bands can be detected (Fig 2B). We detected the myelin protein 2',3'-cyclic nucleotide 3'-phosphodiesterase (CNP) which has been reported as a marker for Schwann cells [23] in both differentiation states. GAPDH served as a loading control. As shown by RT-PCR, undifferentiated as well as differentiated Schwann cells contain *Mbp* mRNA, but sncRNA715 can only be detected in undifferentiated cells (Fig 2C).

These results in cultured Schwann cells imply an inverse correlation of MBP and its inhibitor sncRNA715.

**Fig 3:**
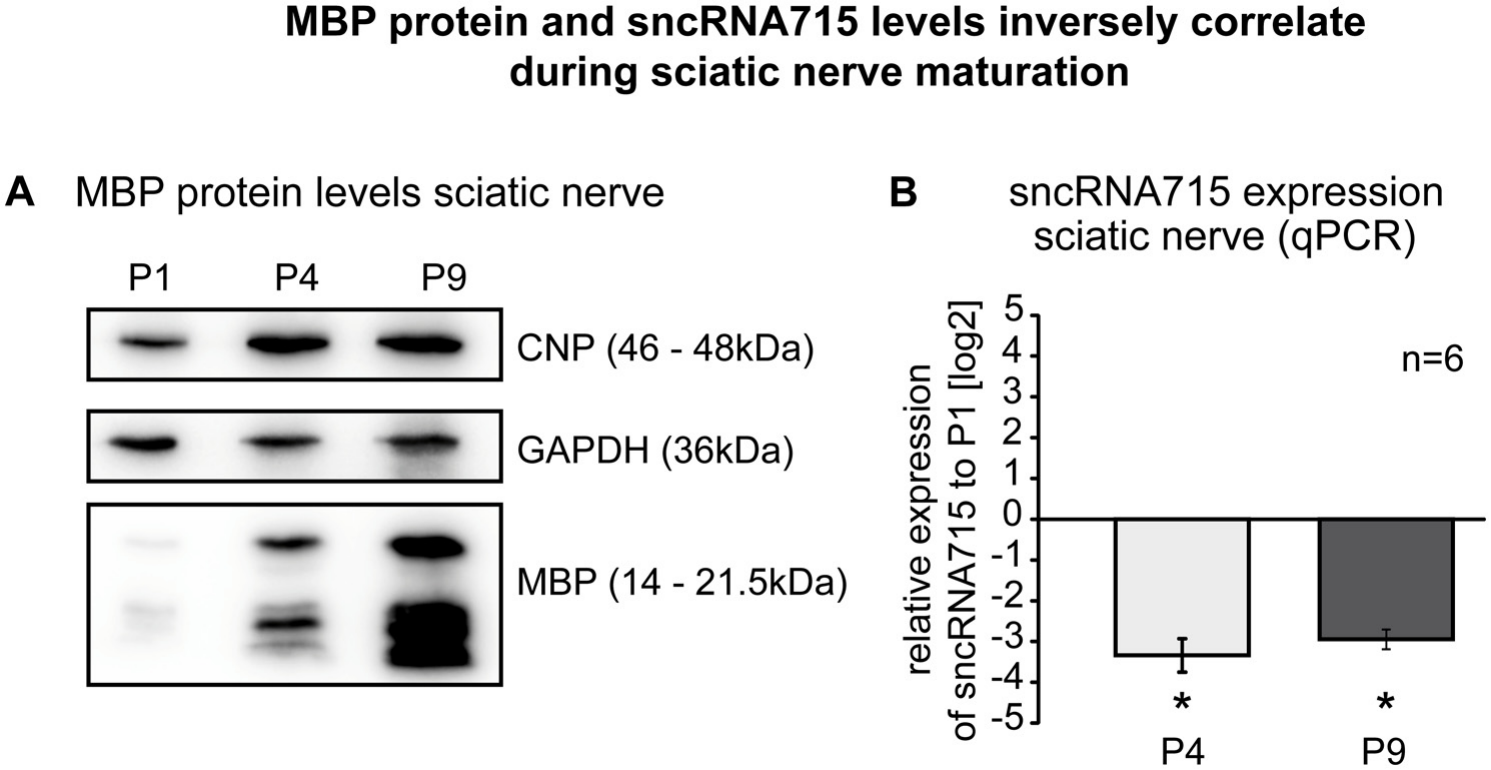
Inverse correlation of MBP and sncRNA715 in the sciatic nerve. **A&B**, The sciatic nerve was lysed from mice at postnatal day 1, 4 and 9 and myelin proteins as well as sncRNA715 expression was analyzed by Western blotting (**A**) and qPCR (**B**), respectively. MBP and CNP Western blots show increasing levels in differentiating sciatic nerves (**A**) while sncRNA715 levels decrease during differentiation, P-values P4: 0,0313, P9: 0,0313 (**B**, log2 values are plotted, sncRNA715 levels at P4 and P9 were quantified relative to P1 using snoRNA135 as a reference gene). Number of experiments (n) are indicated and bar graphs represent mean values ± s.e.m. (Wilcoxon signed-rank test, *P< 0.05, GraphPad Prism5 was used for statistical analysis).

We next assessed if this correlation can also be observed *in vivo*. As shown by Western Blotting, MBP levels increase continuously in the sciatic nerves of mice from postnatal day (P) 1 to 4 and to 9, illustrating the development of the myelin sheath *in vivo* (Fig 3A). CNP levels also increase over time while GAPDH levels (loading control) remain similar at all differentiation stages. We analyzed the sncRNA715 levels in sciatic nerves at the same postnatal days by qPCR and quantified sncRNA715 at P4 and P9 relative to P1 using snoRNA135 as a reference gene. SncRNA715 levels strongly decrease during sciatic nerve development (Fig 3B) from P1 to P4 and remain low at P9. There is no significant reduction of sncRNA715 in the sciatic nerve from P4 to P9 mice.

The inverse correlation of sncRNA715 and MBP in primary Schwann cells and the sciatic nerve support the proposed function of scRNA715 as an inhibitor of *Mbp* translation in the PNS.

### Increasing sncRNA715 levels in differentiated Schwann cells reduces their MBP levels

We next manipulated the levels of sncRNA715 and analyzed potential effects on MBP protein levels.

**Fig 4:**
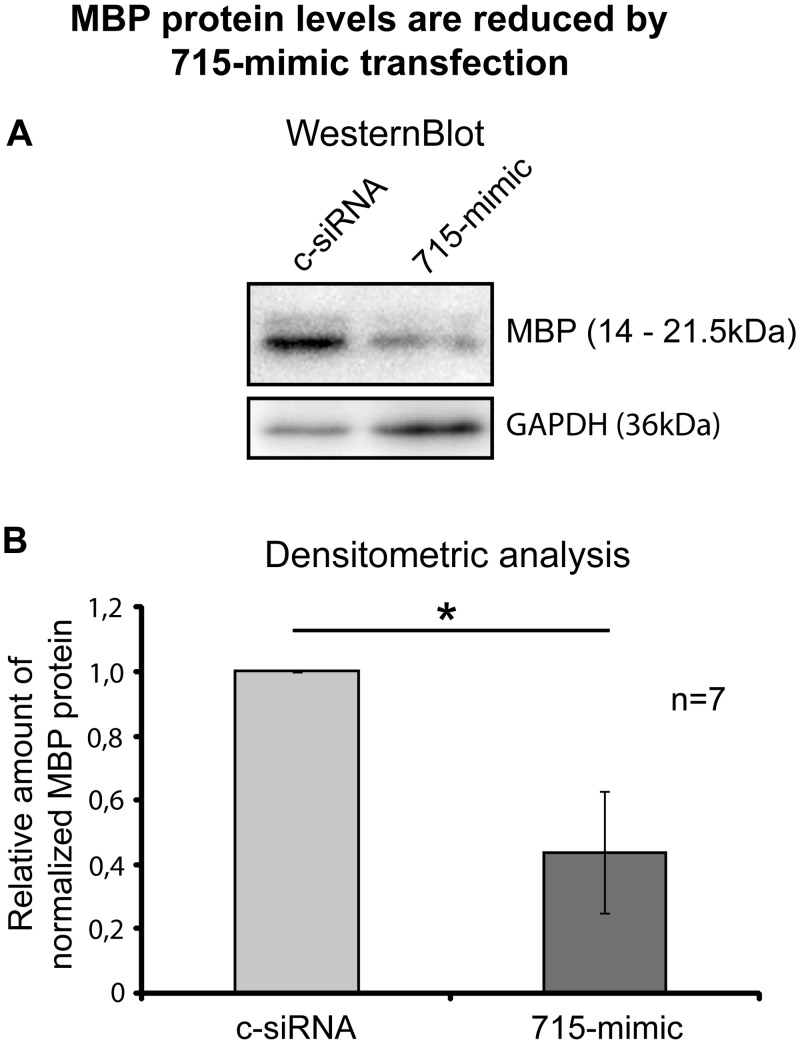
Regulation of MBP levels by sncRNA715. Rat primary Schwann cells were differentiated as described and transfected with synthetic sncRNA715 (715-mimic) or control siRNA (c-siRNA). **A**, Representative Western Blots for MBP and GAPDH showing reduced MBP levels in 715-mimic transfected cells. **B**, Densitometric analysis of 7 experiments (n = 7) as shown in (**A**). Normalized MBP/GAPDH values related to control-siRNA transfected cells are plotted. P-value for 715-mimic: 0.0469. Bar graphs represent mean values ± s.e.m. (Wilcoxon signed-rank test, n = 7, *P< 0.05, GraphPad Prism5 was used for statistical analysis).

Differentiating primary rat Schwann cells were transfected with a synthetic sncRNA715 (715-mimic) or control siRNA. 48 hours post transfection cells were lyzed and MBP and GAPDH (control) levels were analyzed by Western blotting. We observed a strong reduction of MBP protein levels (Fig 4A). We performed 7 independent experiments of this kind and densitometrically quantified the protein bands using the ImageLab Software (Biorad). MBP signals were normalized to GAPDH and the obtained values from 715-mimic-treated cells were related to control siRNA-treated cells. We discovered a significant mean reduction of MBP by approximately 56% in response to 715-mimic transfection (Fig 4B).

These results confirm that sncRNA715 can inhibit the synthesis of MBP in Schwann cells.

## Materials and Methods

### Antibodies, LNA-probes, 715-mimic, primers

Monoclonal antibodies were used against MBP (rat, Serotec, order no. (#) MCA409S) 1:500 in WB and 1:50 in Immunocytochemistry (ICC), CNPase (CNP, mouse, Sigma Aldrich, #C5922) 1:500 in WB and S100 (mouse, Merck-Millipore, clone 15E2E2, #MAB079-1) 1:200 in ICC. Polyclonal antibodies were used against GAPDH (rabbit, Bethyl, #A300-641A) 1:5000 in WB. Secondary antibodies were used from Dianova, (goat anti rat Cy2, #112-225-167) and Life Technologies (goat anti mouse, #A11031).

Double-DIG-labeled 715-specific LNA probe and 3’DIG-labeled U6 control probe used in Northern Blots were purchased from Exiqon (715LNA, 5′CACGCGGGGGTGTGCACGAG3′, order no. 39330–15; U6LNA, 5’CACGAATTTGCGTGTCATCCTT3’, order no. 99002–05). 715-mimic (5′C-UCCGUGCACACCCCCGCGUG3’, order no. MSY0003506) and Allstar control siRNA (order no. 1027280) were obtained from Qiagen. Primers specific for rat MBP: 5′AACATTGTGACACCTCGAACA3′ and 5′TGTCTCTTCCTCCCCAGCTA3′; and for rat glucose-6-phosphate dehydrogenase (G6PDH): 5’TGCAGCAGCTGTCCTCTATG3’ and 5’ACTTCAGCTTTGCGCTCATT3’.

### Cell culture and transfections

IMS32 cells (kindly provided by Kazuhiko Watabe, Tokyo) are spontaneously immortalized murine Schwann cells [21] and they were cultured in DMEM 10% FCS and 4 mM L-Glutamine. Oli-*neu* cells [24] (kindly provided by J. Trotter, Mainz) were cultured on poly-L-lysine in Sato medium (DMEM, 100 μg/l transferrin, 100 μM putrescine, 200 nM progesterone, 500 nM TIT, 220 nM sodium-selenite, 520 mM L-Thyroxine, 0,05% Gentamycine) supplemented with 2% (v/v) horse serum as described before [25].

Primary Schwann cells were extracted from the sciatic nerves of P3 Wistar rats. The nerves were extracted as described earlier [26] and incubated in 0.5% trypsin and 240U collagenase for 90 minutes at 37°C and 5% CO_2_. Digestion was stopped using 1ml FCS in 3ml DMEM and cells were centrifuged at 190x g for 10 minutes. The cells were dissolved in DMEM containing Pen/Strep (100 U/ml), 2mM Glutamax, 10% FCS and cultured for 24h before the medium was exchanged and supplemented with cytosine arabinoside (final concentration 10 μM) to eliminate Fibroblast contamination. After 3 days the medium was removed and expansion medium (DMEM/ F12 1:1, 10% FCS, 4μM Forskolin, 10 ng/ml NRG1-β1 EGF domain) was added.

To induce differentiation and MBP synthesis, rat primary Schwann cells were treated with either a cocktail of 50ng/ml NRG1 and 1mM dbcAMP or 50ng/ml NRG1 and 10μM forskolin. For differentiation the cells were cultured in defined medium (DMEM/ HamF12 (1:1), 0,03% BSA, 100μM putrescine, 100μg/ml transferrin, 11nM progesterone, 400ng/ml T4, 10ng/ml T3, 38ng/ml dexamethasone, 16ng/ml sodium selenite, 0,5% FCS) adopted from [5]. The treatment was performed twice and every second day.

After differentiation cells were transfected with 80 pmol synthetic sncRNA715 (715-mimic) or control siRNA (AllStars Negative Control siRNA, Qiagen) using Lipofectamine RNAiMAX Transfection Reagent according to manufacturer’s protocol (Life Technologies) and after 48 hours protein levels were analyzed by Western blotting.

Primary mouse OPCs were established from C57BL/6 mice postnatal day 9 using the Neural Tissue Dissociation Kit with Papain (Miltenyi Biotec) and Anti-AN2 Microbeads (Miltenyi Biotec) according to manufacturer’s protocol. They were cultured in MACS Neuro Medium containing 1% Pen/Strep, 1% L-Glutamine and 2% NeuroBrew and grown in poly-L-lysine-coated culture dishes.

### Ethics statement

Experiments were performed in accordance with the animal policies of the University of Mainz, approved by the German Federal State of Rhineland-Palatinate, in accordance with the European Community Council Directive of November 24, 1986 (86_609_EEC). Great care was taken to prevent the animals from suffering. Rats and mice were sacrificed by decapitation after isoflurane anesthesia. The experiments were performed under the German animal welfare law (§4) by persons with specific knowledge and skills. All Animals were killed for scientific purposes before using organ material for these studies. A special approval for killing for scientific purposes under §4 of the animal welfare law is not necessary.

### Immunofluorescence and microscopy

Cells were cultured on PLL-coated glass coverslips. Cells were fixed for 5min at room temperature in ice cold 95% Methanol + 5% Acetone and permeabilized with 0.1% (v/v) Triton X-100 in PBS for 2min. After blocking with 10% FCS in DMEM for 15min at room temperature primary antibodies were applied for 1 hour at room temperature. To visualize proteins secondary antibodies conjugated to DyLight488 (1:100) or AlexaFluor568 (1:600) were used in blocking medium for 30min at room temperature. Stained cells were mounted in Mowiol.

Images were acquired with an IX81 microscope with a 40x UPIanFLN (NA = 0.75) objective, a monochrome fluorescence CCD camera XM10 and the cell^F Software (all Olympus).

### Cell lysis and Western Blot

Cells were washed with ice cold PBS and scraped off in lysis buffer (50 mM Tris, 150 mM NaCl, 1 mM EDTA, 1% Triton X-100) containing protease and phosphatase inhibitors (Complete Mini EDTAfree and PhosStop, both Roche Applied Science). Sciatic nerves and whole brains of mice postnatal day 18 (P18) were dissociated in lysis buffer using a TissueRuptor (Qiagen). The lysates were incubated on a rotating wheel at 4°C for 45 min and afterwards cleared from nuclei and debris by centrifugation at 2000x g and 4°C for 5 min.

Separation of proteins were performed by SDS-PAGE using a Mini PROTEAN system (Bio-Rad) or Novex NuPAGE SDS-PAGE Gel system (Life technologies) and transferred onto Roti-PVDF membranes (0.45μm, Roth) using a Mini TransBlot Electrophoretic Transfer Cell device (Bio-Rad). Precision Plus Dual Color Protein Standard (Bio-Rad) was used as a marker. Membranes were blocked with 4% (w/v) milk (Roth) in TBST (50 mM Tris, 150 mM NaCl, pH 7.2, 0.1% (v/v) Tween 20) for 30 min at room temperature. Binding of primary antibodies was carried out either at 4°C over night or for 1h at room temperature. Suitable secondary antibodies (coupled to horseradish peroxidase, Dianova) were incubated for 30min at room temperature. All antibodies were diluted in blocking medium. Bands were analyzed densitometrically using ImageLab Software (Biorad) and MBP Signals were normalized to CNP Signals.

### RNA extraction, reverse transcription, PCR and qPCR

Sciatic nerves were prepared from P 1, 4 or 9 C57BL/6J mice as described above, dissociated in 700μl Qiazol using a Tissue Ruptor (Qiagen) and total RNA was extracted using the miRNeasy Mini Kit (Qiagen). Reverse transcription of mRNAs was performed with the Transcriptor High Fidelity Reverse Transcription Kit and qPCR was performed with the Taqman Universal Master Mix (all Roche Applied Science). SncRNA715 was reverse transcribed by the TaqMan MicroRNA Reverse Transcription Kit with stem-loop RT primers specific for sncRNA715 or snoRNA135 sequence (Applied Biosystems, order no. sncRNA715 PN4427975, snoRNA135 PN440887) and amplified with the Taqman Universal Master Mix (Roche Applied Sience) with specific primers and probes for the indicated sncRNAs (Applied Biosystems). The crossing points were used for relative quantification based on the _ΔΔ_Ct method using REST software [27]. SnoRNA135 was used as a reference gene. PCR products and the 10 bp DNA Step Ladder (Promega) were separated on 4% agarose gels and stained with ethidium bromide.

### Northern Blot

Total RNA from IMS32 cells and undifferentiated primary Schwann cells (2.3 μg each), 10 μl MicroRNA Marker (NEB) and 15 fmol 715-mimic (as positive control) were separated on a 15% Novex TBE-Urea Gel (Life Technologies) and blotted onto a positively charged nylon membrane (Roche Applied Science, #11209299001). Blots were prehybridized for 30 min at 37°C in ULTRAhyb Ultrasensitive Hybridization Buffer (Ambion) and hybridized overnight at 37°C in hybridization buffer containing 1 nM Double-DIG-labeled 715-specific LNA probe or 3’DIG-labeled U6 control probe (both Exiqon) and 10 μl 3′Biotin oligonucleotide Marker probe (New England Biolabs). After 2 washes with 2x SSC for 15 min blots were blocked for 1 h in blocking buffer (100 mM Tris/HCl, 150 mM NaCl, pH 7.5, 1% blocking reagent). Subsequently, blots were incubated for 2 h at room temperature with anti-DIG antibodies conjugated to alkaline phosphatase (AP) (1:5000, Roche Applied Science, #11093274910) to detect hybridized DIG-labeled LNA probe and with anti-Biotin-AP (1:5000, Cell Signaling, #7055) to visualize the marker probe. After 3 washes with Tween-buffer (100 mM Tris/HCl, 150 mM NaCl, 0.3% Tween-20, pH 7.5) and 1 wash with AP-buffer (100 mM Tris/HCL, 100 mM NaCl, 5 mM MgCl_2_), signals were detected using CDP-Star substrate (Roche Applied Science).

## Discussion

The process of myelination must be regulated in a very precise manner including the spatial and temporal control of protein synthesis and maintenance in response to axon-glial contact. Oligodendrocytes myelinate several axonal segments simultaneously. Decentralized synthesis of myelin components in the CNS appears to be a very efficient way to respond rapidly to local variations in axonal properties such as diameter or degree of activity. In the PNS, in which Schwann cells myelinate one axonal segment only, it appears less essential to synthesize myelin components locally in direct response to environmental variations. However, *Mbp* mRNA localization appears to happen in these glial cells, too. This may have evolved due to the basic properties of the protein product which would prematurely compact intracellular membranes and compromise the function of organelles such as the endoplasmic reticulum or Golgi apparatus [28]. The localization of Schwann cell *Mbp* mRNA to myelinating fibres was visualized in trigeminal nerve sections of rats and could be distinguished from *P0* mRNA which remained perinuclearly [13]. Furthermore, *Mbp* mRNA could be detected diffusely in the Schwann cell internode and predominantly in the paranodal cytoplasm [14]. These findings strongly suggest that *Mbp* mRNA is transported into Schwann cell processes similar to oligodendroglial *Mbp* [15], but the transport mechanisms have not been elucidated so far. It is likely that, as in oligodendrocytes, *Mbp* mRNA is sorted into RNA-granules by binding to the trans-acting factor hnRNP A2 and transported along the cytoskeleton to the axon-glial contact site.

Although mRNA trafficking mechanisms in Schwann cells remain to be investigated in detail, it is clear that *Mbp* mRNA is translationally silenced and our expression data in IMS32 and primary Schwann cells support this. We furthermore confirmed that Schwann cell MBP synthesis is inhibited by the small non-coding RNA 715. In addition to transcriptionally controlled upregulation of *Mbp* and other myelin mRNAs during Schwann cell differentiation, this posttranscriptional mechanism appears to add an additional level of synthesis control. Mutational analysis of the sncRNA715 binding site in *Mbp* mRNA has demonstrated in luciferase reporter assays that sncRNA715 binds to a distinct sequence in the 3’UTR and thereby represses translation of *Mbp* mRNA [16]. Interestingly, it was shown in the CNS that sncRNA715 levels are abnormally high in chronic multiple sclerosis lesions indicating a possible role as an inhibitor of remyelination events during the course of disease [16].

SncRNA715 was initially classified as a microRNA, but was then excluded from the microRNA Database miRBase [29] because the deep sequencing reads were inconsistent with miRNA processing. It appears that a number of sncRNAs including sncRNA715 originate from ribosomal DNA (rDNA) and were termed small rDNA-derived RNAs (srRNAs) [30]. The 18S, 5,8S and 28S ribosomal RNAs (rRNAs) are processed from a large 47S RNA polymerase I transcript. Interestingly approximately half of this 47S rRNA precursor consists of the 5’ and 3’ externally transcribed spacer (ETS) and the internally transcribed spacer (ITS) 1 and 2. The sncRNA715 sequence is located in the large 5’ETS and is part of a 19S fragment which accumulates during rRNA precursor processing [31]. It remains to be shown how sncRNA715 or other srRNAs are processed from rRNA precursor molecules and how this results in different levels of sncRNA715 during cell differentiation or in different cell types which we observed previously [16].

The importance of sncRNA function for Schwann cell development was initially suggested by the finding that the expression of PMP22, a protein which is involved in Schwann cell differentiation, is regulated by miRNA-29a [18]. Furthermore, the interference of miRNA synthesis by knock down of the Dicer ribonuclease resulted in decreased levels of MBP and P0 as well as a reduction of myelin synthesis in myelinating dorsal root ganglion cocultures [32]. A strongly hypomyelinated PNS and increased Schwann cell apoptosis as well as proliferation was observed in mice in which Dicer was ablated specifically in Schwann cells [19]. Schwann cells lacking Dicer seem to remain in a promyelinating differentiation state being incapable of synthesizing myelin [20]. An involvement of Dicer in the synthesis of sncRNA715 has not been investigated so far.

After PNS nerve injury intrinsic Schwann cells are capable of dedifferentiating by changing their transcriptional profile and actively contribute to the regenerative process [33]. MiRNA profiling after nerve injury in the presence or absence of Dicer revealed an involvement of these sncRNAs in the required expression changes inducing a regenerative response [34]. Proteins like MBP, P0 and PMP22 are downregulated after nerve injury [35] and it would be interesting to analyze the levels and the possible role of sncRNA715 during the process of regeneration. In particular the question arises if inhibition of endogenously expressed sncRNA715 could boost remyelination of regenerating nerves.

In summary, we found sncRNA715 expressed by myelinating cells of the PNS, where it regulates Myelin Basic Protein synthesis. Together with former findings in oligodendrocytes [16] we suggest a general regulatory role of sncRNA715 in controlling translational inhibition of *Mbp* mRNA during transport to the site of myelination, where local translation takes place.
